# Clinical performance evaluation of the Idylla™ *EGFR* Mutation Test on formalin-fixed paraffin-embedded tissue of non-small cell lung cancer

**DOI:** 10.1186/s12885-020-6697-7

**Published:** 2020-04-03

**Authors:** Mercedes Delgado-García, Birgit Weynand, Lourdes Gómez-Izquierdo, María José Hernández, Ángela María Blanco, Mar Varela, Xavier Matias-Guiu, Ernest Nadal, Bélgica Márquez-Lobo, Ana Alarcão, Enrique de Álava, Michele Biscuola

**Affiliations:** 1 Department of Pathology, Molecular Pathology Laboratory, Hospital Universitario Virgen del Rocío-IBIS, Av. Manuel Siurot, S/n, 41013 Sevilla, Spain; 2 Department of Pathology, Universitair Ziekenhuis Leuven, Leuven, Belgium; 3 Department of Pathology, Hospital Universitari de Bellvitge, Idibell, Oncobell, Barcelona, Spain; 4 Department of Pathology, Hospital Universitari Arnau de Vilanova, University of Lleida, IRBLleida, CIBERONC, Lleida, Spain; 5 Department of Medical Oncology, Catalan Institute of Oncology, Idibell, Oncobell, Barcelona, Spain; 6 Department of Pathology, Complejo Hospitalario de Jaén, Jaén, Spain; 7 Institute of Anatomical and Molecular Pathology, Faculty of Medicine, University of Coimbra, Coimbra, Portugal

**Keywords:** Non-small-cell lung carcinoma, *EGFR*, Mutations

## Abstract

**Background:**

Detection of epidermal growth factor receptor (*EGFR)* mutations in exons 18–21 is recommended in all patients with advanced Non-small-cell lung carcinoma due to the demonstrated efficiency of the standard therapy with tyrosine kinase inhibitors in *EGFR*-mutated patients. Therefore, choosing a suitable technique to test *EGFR* mutational status is crucial to warrant a valid result in a short turnaround time using the lowest possible amount of tissue material.

The Idylla™ *EGFR* Mutation Test is a simple, fast and reliable method designed for the detection of *EGFR* mutations from formalin-fixed paraffin-embedded samples.

The aim of this study was the Clinical Performace Evaluation of the Idylla™ EGFR Mutation Test on the Idylla™ System.

**Methods:**

*EGFR* mutational status was determined on 132 archived formalin-fixed paraffin-embedded tissue sections with Idylla™ technology. Results were compared with the results previously obtained by routine method in the reference lab (Therascreen® *EGFR* RGQ PCR v2, Qiagen in Molecular Pathology lab, Hospital Universitario Virgen del Rocío de Sevilla).

**Results:**

The overall agreement between results obtained with the Idylla™ *EGFR* Mutation Test and the Comparator test method was 95.38% (with 1-sided 95% lower limit of 91.7%) showing Positive Diagnostic Agreement of 93.22% and Negative Diagnostic Agreement of 97.18%, with a Limit Of Detection ≤5%.

**Conclusions:**

The Idylla™ *EGFR* Mutation Test passed its clinical validity performance characteristics for accuracy.

## Background

Non-small-cell lung carcinoma (NSCLC) is one of the most common cancers worldwide, contributing for 13% of all cancer types [1]. NSCLC represent the 85% of lung cancer, mainly subdivided into two types: squamous cell carcinoma (SCC) and non-squamous cell carcinoma (non-SCC) of which histologically adenocarcinoma (ADC) is the most prevalent one (50%) [2, 3].

Mutations in the *EGFR* gene are commonly observed in NSCLC particularly in ADC [4]. The gene encodes a transmembrane glycoprotein, EGFR or HER1/ErbB1, member of the epidermal growth factor tyrosine kinase (TK) receptors’ family ERbB. Binding of EGFR to its ligands causes dimerization and tyrosine autophosphorylation. Downstream cascade activation of pathways, results in cellular proliferation and survival [5, 6]. *EGFR* gene mutations, increased gene copy number and overexpression of EGFR proteins can however lead to constitutive TK activity and carcinogenesis [7].

*EGFR* mutations are present in around 15% of NSCLC [8]. *EGFR* mutation testing for activating “hot-spot” mutations in exons 18–21 is recommended in all patients with advanced NSCLC of the non-SCC subtype [9]. Exon 19 deletions, exon 21 (L858R, L861Q), and exon 18 (G719X) mutations are associated with sensitivity to *EGFR*-tyrosine kinase inhibitors (TKIs) [10–12] where exon 21 L858R point mutation and exon 19 deletions are the most frequent alterations (overall 85–90%) [13]. Exon 20 (S768I) confers a good outcome to first generation *EGFR* TKIs (erlotinib, gefitinib), although its association with sensitivity or resistance to TKIs is still not known [14]. Exon 20 insertions may predict resistance to TKIs. Moreover, *EGFR* T790M mutation is one of the main causes of acquired resistance to TKI therapy and has been reported in about 55% of patients with disease progression after initial response to 1st or 2nd generation TKIs [10, 11]. C797S is a second acquired resistance mutation, arising in tumors that have progressed after (osimertinib) treatment for T790M+ disease. Nevertheless, this mutation is unusual and not currently treatable [15].

Various commercial assays are used in routine practice to test the presence of *EGFR* mutations in a tumoral context and the majority of those have been optimized to be compatible with DNA extracted from formalin-fixed paraffin-embedded (FFPE) samples (obtained from diagnostic biopsies, surgical resections and even cytological specimens). Each assay is characterized by a specific range of covered mutations, different level of automation and multiplexing, variable cost, high turnaround time plus the need for specialized equipment and highly skilled staff but most of all, each of them is characterized by specific sensitivity, specificity and Limit of Detection (LOD) for each one of the tested mutations.

The Idylla™ *EGFR* Mutation Test, as the Idylla™ *KRAS* and *BRAF* Mutation Tests, is a fully-automated real-time-PCR-based test designed for the detection of *EGFR* mutations in a quick turnaround time (approx. 150 min) from FFPE sample to final result. The test is performed directly on one FFPE tissue section, requiring no beforehand sample preparation and DNA extraction and minimal hands-on time. The interpretation of results is fully automated.

This study was conducted as a Clinical Performance Evaluation (CPE) to evaluate the performance of the Idylla™ *EGFR* Mutation Test on the Idylla™ System under the conditions of use [16, 17], in accordance with the quality standard EN-13612 (2003) ‘Performance evaluation of in vitro diagnostic medical devices’, in order to demonstrate the followings objectives: a) overall, positive and negative agreement for sensitizing and resistant mutations; b) positive and negative agreement at the specific genotype call level; c) positive and negative diagnostic agreement of at least 90% of the Idylla™ *EGFR* Mutation Test by comparing it to a comparator test (Therascreen® *EGFR* RGQ PCR v2, Qiagen), in tumor samples of subjects with NSCLC.

## Methods

### Patients and samples

**Fig. 1:**
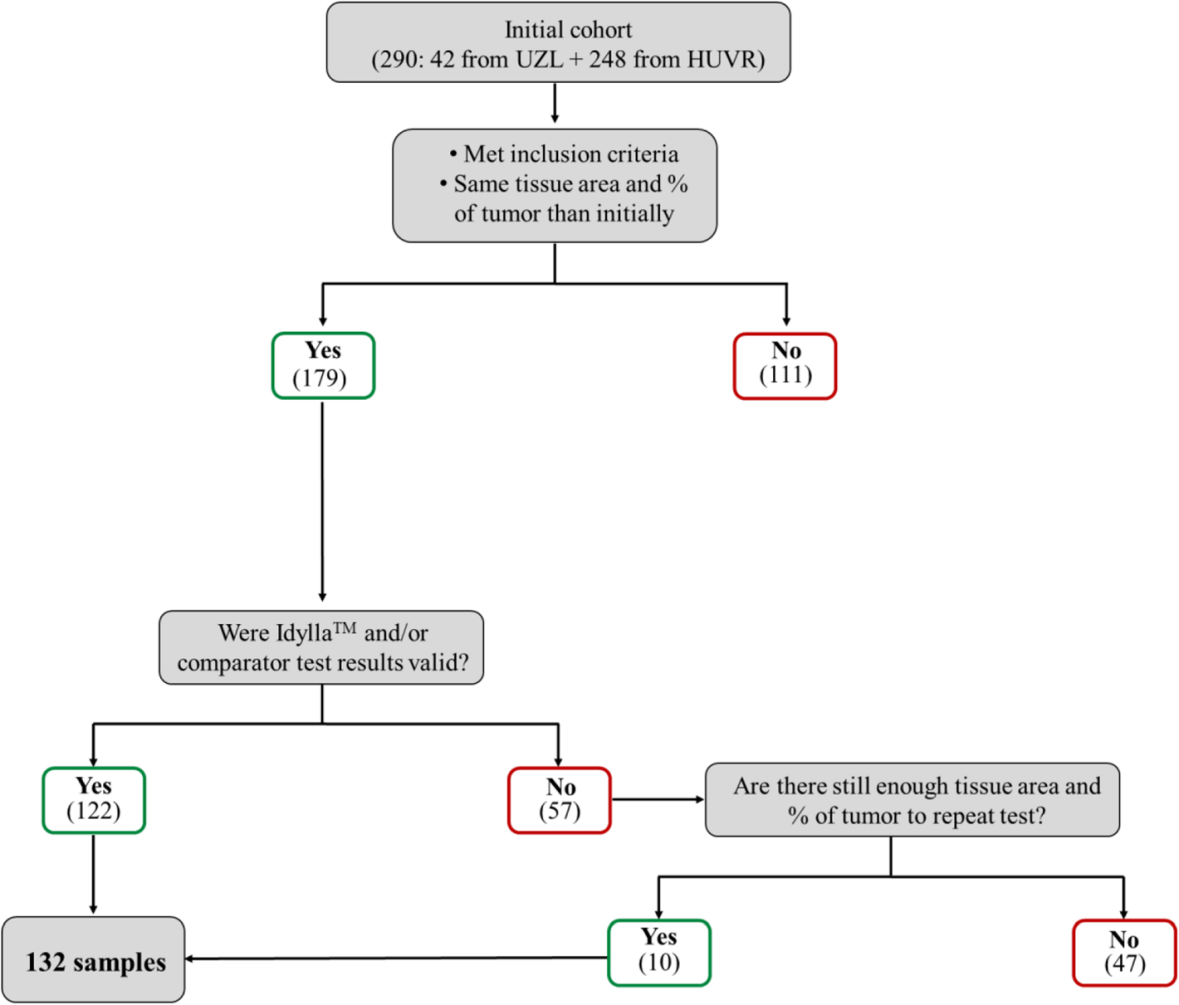
The sample selection process

Samples were recruited from four different centers [Hospital Universitario Virgen del Rocío de Sevilla, Spain (HUVR), Universitair Ziekenhuis Leuven, Belgium (UZL), Complejo Hospitalario de Jaén, Spain (CHJ) and Hospital Universitario de Bellvitge, Spain (HUB)]. The CPE study was carried out at two sites: UZL and HUVR (reference lab due to accreditation under the UNE-EN ISO 15189:2013 related with this technique). Samples used for this study were slides or curls (slices) from archived, appropriately stored and adequately identified FFPE tumor blocks. The initial study cohort consisted of 290 samples (42 from UZL and 248 from HUVR). UZL tested 42 samples with Idylla™ *EGFR* Mutation test and no samples with Therascreen V2. HUVR tested 137 samples with Idylla™ *EGFR* Mutation test and 179 samples with Therascreen V2. We excluded samples due to: a) inclusion criteria not met (Table 1); b) absence of valid results for both test; c) insufficient material (comparation of an initial hematoxylin with the final one). Attending to this, the final analysis was performed on 132 samples (Fig. 1) (Table 2).

**Table 1:** Inclusion criteria for the Idylla™ test

	Inclusion criteria
**1**	Male or female patients ≥18 years of age
**2**	Samples can be used for investigational purposes according to the applicable laws
**3**	Histological confirmed primary or metastatic NSCLC with known *EGFR* status (valid result with the version 2 of the comparator test)
**4**	For Idylla™ testing: one slice or slide with a minimum of 10% tumor cells of the total tissue used (if this is not obtained, macro-dissection is to be performed to reach at least 10% tumor cells in total tissue area used)
**5**	FFPE blocks from the institute, which preferably had a maximum fixation time of 48 h (routine procedure) and are preferably not older than 5 years after the date of collection, stored at ambient conditions

**Table 2:** CPE study cohort

CPE study	Positive ***EGFR***	Negative ***EGFR***	Total
Sevilla	27	47	74
Jaen	6	0	6
Leuven	19	22	41
Bellvitge	11	0	11
**Total**	**63**	**69**	**132**

Patients provided informed consent for investigational purposes and the institutional ethics committees of all these centers approved the study. Main features of the patients are shown in Table 3.

**Table 3:** Demographic and clinical characteristics of patients. * Mean age (year) at tumor collection date

Total number (NSCLC)	132
Gender	Male: 56% Female: 43% Missing: 1%
Mean age (year)*	65
	Sd: 10.12 median: 65
	min: 44 max: 85
	Missing: 11
**Tissue location**	**Number of patients (%)**
Primary	85 (64%)
Distant metastases	15 (11.5%)
Metastasis in lymph nodes or pleura	15 (11.5%)
Unknown	17 (13%)

Once CPE study was finished, the reference lab included 82 extra samples [from HUVR, HUB, Hospital Universitario Mútua Terrassa, Spain (HUMT) and Facultade de Medicina da Universidade de Coimbra, Portugal (FMUC)]. After the exclusion of 29 of these samples for the same reasons as described earlier, 53 samples were compared for Idylla™ and comparator test (supplementary Table S1).

Five-μm thick FFPE tissue sections were prepared as close as possible to the sections previously used to generate the reference results. Tumor content, percentage of necrosis, presence/absence of TAR (a large variety of organic and inorganic chemicals generated by burning tobacco that forms a brown substance between lung cells; it is the main cause of lung and throat cancer in smokers.) and area were determined on a hematoxylin-eosin (HE)-stained slide by a pathologist. Macro-dissection was performed to achieve tumor cell content of at least 10%.

### Therascreen® *EGFR* RGQ PCR kit version 2 used as reference method

Therascreen® *EGFR* RGQ PCR Kit (version 2) was performed according to the manufacturer’s instructions. This is an In-Vitro Diagnostic (IVD) test for the detection of 29 somatic mutations G719A/S/C in exon 18, 19 deletions in exon 19, T790M, S768I and 3 insertions in exon 20, and L858R, L861Q in exon 21 in the *EGFR* oncogene, using Scorpions® and ARMS® technologies in real-time PCR. The Therascreen® *EGFR* RGQ PCR Kit was tested on DNA samples extracted from FFPE tumor tissue from NSCLC patients (Qiagen QIAamp® DNA FFPE-kit), and run on a Rotor-Gene Q MDx instrument.

### Idylla™ *EGFR* mutation test

The Idylla™ *EGFR* Mutation Test used in the study was an investigational use only labeled product as the IVD version was at that moment not yet commercially available. This was the same product as the IVD version except for its labeling. The Idylla™ *EGFR* Mutation Test is a test for the qualitative detection of 51 *EGFR* mutations: exon 18 (G719A/S/C), 36 deletions in exon 19 (the set of 36 mutations in exon 19 detected with Idylla™ fully overlap the 19 mutations detected with Therascreen), exon 20 (T790M, S768I), 5 insertions in exon 20 (the 2 sets of mutations are overlapping in 2 mutations (c.2310_2311insGGT; p.D770_N771insG and c.2319_2320insCAC; p.H773_V774insH).) and exon 21 (L858R, L861Q) in the *EGFR* oncogene in FFPE human malignant lung cancer tissue.

FFPE tissue sections were placed (one per sample) directly into the cartridge of the fully automated Idylla™ platform (Biocartis, Mechelen, Belgium) following the manufacturer’s instructions, without requiring prior manual deparaffinization or FFPE pre-processing. With a hands-on time of less than 2 min and a total turnaround time of 150 min, the instrument covers fully integrated sample preparation (with a combination of reagents, enzymes, heat, and high intensity focused ultrasound (HIFU) inducing deparaffinization, disruption of the tissue, and lysis of the cells) combined with PCR thermocycling (via microfluidic channels in the cartridge, nucleic acids are transported into 5 separated chambers with dried form PCR reagents) and fluorescence detection of target sequences, using allele specific primers. A sample processing control (SPC) is included in each run and the presence of a mutant genotype is determined by calculating the ΔCq (quantification cycle) between the *EGFR* SPC and the *EGFR* mutant signal(s). All required consumables are provided in the cartridge and the Idylla™ Console and the Idylla™ instruments are CE marked.

### Evaluation of samples and interferences

Although inclusion criteria were well established, an assessment was made for different characteristics of samples to avoid invalid or false results, including:

i) Age of prepared FFPE blocks: 14 samples with an unknown preparation date and 9 blocks older than 5 years.
ii) Macro-dissection: needed to increase the percentage of tumor nuclei to reach at least 10%.
iii) Tissue area: tissue area of samples was between 1 and 567 mm^2,^ since there was no minimum tissue area requirement input for the *EGFR* Mutation test.
iv) Other interferences: the presence of necrotic tissue and TAR.

Nevertheless, if an invalid result was obtained, both tests were repeated once. Invalid results may be caused by a variety of reasons including presence of inhibitors in the sample, insufficient DNA, incorrect placement of a sample in a cartridge and/or sample volume out of range. At this point, it is important to claim that the repetition for invalid results in Therascreen was part of the study, but it is also part of our routine diagnostic protocol, usually modifying DNA concentration and/or repeating extraction in order to avoid necrosis or TAR.

### Analysis of discordant results

A third method was used to further analyze some of the samples having an Idylla™ *EGFR* Mutation Test result not concordant with the result of the reference method. Next generation sequencing (NGS) and/or Droplet Digital™ PCR (ddPCR) were used depending on the quantity of leftover material available (sections close to those used for the other tests were provided).

NGS was done (with a minimum amount of 8 slices) by a validated workflow of the Tumor Hotspot MASTR™ Plus kit (Multiplicom) on the Illumina MiSeq Dx instrument. NGS and the subsequent data-analyses pipeline was done by Histogenex (minimal total mean read depth of 185.000, exon coverage of 500x mean read depth).

The ddPCR was performed at Biocartis. ddPCR was done on liquefied FFPE material using commercially available ddPCR assays (Droplet Digital™ PCR Assays and QX200 ddPCR system, Bio-Rad Laboratories, Inc.). These predesigned assays contain probes for the detection of both WT and all the specific mutations. Samples were considered positive by ddPCR when the % mutant was ≥1%, except for T790M which was considered positive when the % mutant was ≥5%.

Furthermore, it was necessary to analyze the degree of fragmentation to see if the DNA in the discordant samples was heavily fragmented or not, which could be a problem for a PCR based analysis method like Idylla™. A 5-plex PCR was developed and executed by Biocartis. Samples were liquefied on the platform following a PCR reaction for 5 housekeeping genes: β-actin (321 bp), ABCB (213 bp), TFRC (149 bp), HPRT (105 bp) and RNaseP (63 bp). A sample is considered fragmented when the size of the amplicons detected with the 5-plex PCR is smaller than the amplicons that would be needed for the *EGFR* test (*EGFR* PCR products range from 67 to 170 bp).

### Statistical analysis

Ninety-five percent two sided confidence interval based on Wilson’s score method [18] at the dichotomous level (“mutation detected” versus “no mutation detected”) was used for the estimation of total, positive and negative agreement.

Specificity and sensitivity were defined as the proportion of concordant results against the sum of concordant and discordant results (true positives / (true positives + false negatives) and true negatives / (true negatives + false positives). Analyses were performed in R software 3.2.5 (R Core Development Team, 2016).

## Results

### Comparator test and Idylla™ *EGFR* mutation test

In a primary analysis on dichotomous level, the invalid runs at first testing were excluded, resulting in a total of 122 samples. After repeat testing, 10 more samples provided a valid result for Idylla™ and/or Comparator test V2, leading to a total of 132 samples included for the secondary analysis.

The *EGFR* mutational status of 132 retrospective clinical FFPE samples from patients with primary or metastatic NSCLC was tested with Idylla™ System (Idylla™), and results were compared with the original assessments made by Therascreen® *EGFR* RGQ PCR Kit (version 2). Idylla™ results were not used for any diagnostic or therapeutic purposes.

We obtained 57 positive samples for *EGFR* mutation and 75 wild-type cases. Idylla™ demonstrated agreement with routine method in 121 out of 132 samples (91.7%). Two samples had an Idylla™ result with a positive mutation detected that was not detected by the Therascreen® test. Four samples had a Therascreen® result with a positive mutation that was not detected by the Idylla™ test. Three samples had different positive mutations detected by both tests, and two samples contained a specific exon 20 insertion: c.2311_2319dupAACCCCCAC; p.Asn771_His773dup [p.N771_H773dup] that was not targeted by the Idylla™ *EGFR* Mutation Test and were therefore discordant by design (Table 4).

**Table 4:** Agreement table at the mutation specific level

	Therascreen®									
Idylla™	DelEx19	DelEx19,T790M	L858R	L861Q	G719X	G719X, S768I	L858R,T790M	InsEx20	WT	Totals
DelEx19	20									**20**
DelEx19,T790M		5								**5**
L858R			22				1			**23**
L861Q				2						**2**
G719X					1					**1**
G719X,S768I						1				**1**
L858R,T790M							1			**1**
T790M		1							1	**2**
DelEx19, S768I	1									**1**
S768I									1	**1**
WT		1	2		1			2	69	**75**
**Totals**	**21**	**7**	**24**	**2**	**2**	**1**	**2**	**2**	**71**	**132**

### Samples characteristics

The age of the FFPE blocks should be preferably maximum 5 years after the date of collection. This was the case for all the blocks except for 14 samples for which the preparation date is “unknown” and 9 blocks which were older than 5 years. All samples have been included in the analysis, since no age-related trend regarding invalid tests was identified (supplementary Table 2).

Tissue area of samples was between 1 and 567 mm^2^. No tissue size-related trend was observed in valid and discordant rate. The samples with a tissue area below 10mm^2^ still led to valid and concordant results in > 90% of the samples (supplementary Table 3).

The influence of the presence of necrotic tissue and TAR on the results was evaluated in all samples where possible. Results showed that neither necrotic tissue-related nor TAR-related trends were apparent in the obtained results (supplementary Table 4).

### Comparison between both methods

A valid result with both methods was obtained for 132 out 179 of initially selected samples. After the exclusion of two discordant results by design, the agreement between Idylla™ and the comparator test was calculated based on the dichotomous response whether a mutation was detected or not. Table 5 shows the raw data used to calculate it. The overall agreement (defined as the proportion of concordant results in all results) for 130 samples was 95.38% with a lower limit of the 95% confidence interval (CI) of 91.32. Positive agreement was calculated to be 93.22% CI of 85.73, and, the negative agreement was calculated to be 97.18% CI of 91.84 (Table 6).

**Table 5:** Agreement table at the dichotomous level for valid, non-missing results

	Therascreen®		
Idylla™	Mutant	WT	Totals
Mutant	55	2	57
WT	4	69	73
Totals	59	71	**130**^**a**^

^a^*n* = 130 samples after excluding two discordant by design

**Table 6:** Measures of agreement

Measure	Rate	Point estimate	95% lower limit (1-sided)	95% upper limit (1-sided)
Overall Diagnostic Agreement	124/130	95.38	91.32	100
Positive Diagnostic Agreement	55/59	93.22	85.73	100
Negative Diagnostic Agreement	69/71	97.18	91.84	100

Invalid results were obtained for 47 out 179 selected samples: with Idylla™ or with comparator test or with both technologies: invalid result only for Therascreen® (34 out 47); invalid result only for Idylla™ (6 out 47); invalid result for both tests (7 out 47).

An additional post-CPE study was done with 53 extra samples with the same characteristics described previously. Idylla™ results showed agreement with routine method in 49 samples. We found that 3 samples had a Therascreen® result with a positive mutation detected that was not detected by the Idylla™ test, and 1 sample had different positive mutations detected by the two tests. However, the overall concordance between the Idylla™ *EGFR* Mutation Test and the reference routine method was found to be 94.34%, with a negative agreement of 100% and a positive agreement of 89.26% (supplementary Tables 5, 6 and 7). Discordant results were not analyzed.

### Discordant results

Discordant samples were tested with NGS and/or ddPCR to investigate the root-cause of the discordances.

A discordant result was observed for 11 samples (Table 4). Two discordant results were the result of a specific insertion (p.Asn771_His773dup) that is not targeted in the design of the Idylla™ *EGFR* Mutation Test (discordant by design) and were not taken into account in the agreement calculations. Four discordant samples had insufficient material for NGS analysis. The remaining five discordant samples were included in NGS analyses, together with six random concordant samples (Table 7). All six concordant samples were confirmed using NGS (data not shown).

**Table 7:** Results of discordance analysis and conclusions of the root cause

	Therascreen® V2	Idylla™	NGS	ddPCR	Fragmentation	Result/ Root case
**1**	Wild type	T790M	T790M	Wild type for T790M	No	Confirmed with NGS
**2**	Wild type	S768I	ND^a^	ND^a^	ND	No leftover material for analysis
**3**	G719X	Wild type	G719X^b^	low input	Yes	Sample quality (fragmentation) + low sample input
**4**	DelEx19	Wild type	p.Glu746_Al a750del	low input	No	Low sample input + low allelic frequency in NGS (6%)
	T790M		wild type	low input		Low sample input
**5**	L858R	Wild type	L858R	low input	No	Low sample input
**6**	L858R	Wild type	ND^a^	low input	yes	Sample quality (fragmentation) + low sample input
**7**	L858R	L858R	ND^a^	low input	No	Low sample input, insufficient material for NGS
	T790M			wild type for T790M		
**8**	DelEx19	DelEx19	DelEx19	DelEx19 not tested	No	Concordant result
	Wild type	S768I	wild type	6,7% S768I		Low allelic frequency (as confirmed with ddPCR)
**9**	DelEx19	T790M	ND^a^	Wild type for Del15 & Del 18	No	Confirmed with ddPCR, insufficient material for NGS
	T790M			6,2% T790M		

^a^ Not enough material available; ^b^ Variant Allele Frequency below LOD and present in only one of two amplicons covering the variant position; *ND* Not determined

## Discussion

Molecular diagnosis for activating “hot-spot” mutations in *EGFR* exons 18 to 21 is recommended in all patients with advanced NSCLC of a non-SCC subtype since TKI therapy provides significant improvement in survival and quality of life [9].

Classical methods for testing *EGFR* in FFPE material require tissue deparaffinization, manual isolation of DNA and DNA quantification [9]. Therascreen® *EGFR* RGQ PCR Kit (version 2) with a total turnaround time of 20 h (including DNA extraction, purification and PCR), is considered a robust method for the detection of ‘hot spot’ mutations predictive of TKI response, and is widely utilized [19, 20].

The Idylla™ *EGFR* Mutation Test, performed on the Biocartis Idylla™ System, is an in vitro diagnostic fully automated real-time PCR based test for the qualitative detection of *EGFR* mutations, from FFPE sample to final result. The test is performed directly on one FFPE tissue section, requiring no beforehand sample preparation and minimal hands-on time, yielding results within 2.5 h. This test encompasses 51 *EGFR* clinically relevant mutations from exons 18 to 21 in human lung cancer FFPE material (according to the latest IASLC atlas of *EGFR* testing in lung cancer) [21].

The performance of the Idylla™ platform has been previously reported in various studies [16, 17, 22–25].

This CPE study compared the Therascreen® *EGFR* RGQ PCR v2 to the Idylla™ *EGFR* Mutation Test, showed an overall presence of *EGFR* mutations in the samples of 45.38% when measured with Therascreen® *EGFR* RGQ PCR v2 and of 43.85% measured with the Idylla™ *EGFR* Mutation Test, demonstrating that the sensitivity of the Idylla™ *EGFR* Mutation Test is comparable to the sensitivity of the routine reference method. The overall concordance between the Idylla™ *EGFR* Mutation Test and the reference routine method was found to be 95.38% [95% CI: 91.32%-100], indicating a good concordance, showing the near-equivalence of both measuring techniques, and, consequently, test robustness for Idylla™.

After the exclusion of 2 results discordant by design (with the specific insertion p.Asn771_His773dup that is not targeted in the design of Idylla™ *EGFR* Mutation Test), 9 discordant results were found between both methods. Using NGS and/or ddPCR and analyzing the degree of DNA fragmentation, the main reasons of these discrepancies were studied, including insufficient material, low sample input and/or low allelic frequency (in the cases with enough leftover material and good sample quality) (Table 7).

Besides, several other pre-analytical parameters were evaluated, such as the age of the FFPE blocks, tissue area, percentage of necrotic tissue, and the presence of TAR. For these 4 parameters, no correlation could be found, indicating that the Idylla™ *EGFR* Mutation Test is a robust test.

The number of invalid results, obtained with one of the two methods or with both, represents an interesting aspect of this study. We obtained a Therascreen® invalid result for 34 out 47 samples. As we detailed before, repetitions with this test occur during our routine practice and, for those cases, we usually modify DNA concentration and/or repeat extraction in order to avoid necrosis or TAR. However, although we modified the starting material in the repetitions, this strategy was not enough to obtain an evaluable result for these samples tested with the comparator test.

Invalid results for Idylla™ (6 out 47 samples) were mainly due to the absence of amplification of the internal control. We repeated the test at least one time for each one of the invalid cases obtaining the same result. We considered to try to avoid necrosis or TAR with macrodissection but, as previously demonstrated, these characteristics do not interfere with the final result.

Finally, invalid results for both test were obtained for 7 out 47 samples, and, although we repeated these samples with both techniques once, we obtained the same result. We argued that this kind of samples presented some intrinsic artefacts no controlled in our lab, for example, an improper fixation time and/or a more elevated presence of specific PCR inhibitors due to the process, concluding that the quality of these samples was not adequate to study the mutational status of the *EGFR* gene.

The small number of invalid samples obtained with Idylla represents an important aspect for the *EGFR* status determination because more patients could be screened for these mutations in order to receive a more personalized treatment.

The advantages of this system have been clearly exposed before [16, 17, 22–25], the load of FFPE samples directly into single use cartridges with minimal sample preparation (and as consequence, minimal probability of contaminations), a quick turnaround time and a fully automated interpretation of results. Some disadvantages and/or cautions need to be kept in mind, such as a limited throughput (due to the system only processing one sample at a time) and, consequently, the difficulties for laboratories with high daily sample workload. However, is possible to add up to eight different modular systems to the same console.

At this point, it is important to emphasize the crucial importance of the pathologist’s pre-analytical evaluation in assessing the percentage of tumor cells, and other characteristics. Previous (and mandatory) evaluation of tissue helps to avoid false and/or invalid results.

## Conclusions

All the characteristics exposed before, together with the high concordance with the reference method [Therascreen*® EGFR* RGQ PCR Kit (version 2)], indicate that Idylla™ *EGFR* Mutation Test on the Idylla™ System is a fully-automated method, designed for the detection of *EGFR* mutations in a quick turnaround time from FFPE sample to final result and can be a suitable clinical test for routine use in diagnostic procedures.

## Supplementary information

**Additional file 1 Supplementary Table 1.** post-CPE study cohort. **Supplementary Table 2.** age of prepared FFPE blocks. **Supplementary Table 3**. assessment of the tissue area. **Supplementary Table 4.** evaluation of necrotic tissue and tar. **Supplementary Table 5:** post-CPE results. **Supplementary Table 6.** agreement table at the dichotomous level for valid, non-missing results. **Supplementary Table 7.** post-CPE measures of agreement.

## Data Availability

The datasets used and/or analyzed during the current study are available from the corresponding author upon reasonable request.

## Abbreviations

ADC: Adenocarcinoma
CPE: Clinical performance evaluation
ddPCR: Droplet digital™ PCR
*EGFR*: Epidermal growth factor receptor
FFPE: Formalin-fixed paraffin-embedded
HE: Hematoxylin-eosin
HIFU: High intensity focused ultrasound
IVD: In-vitro diagnostic
LOD: Limit of detection
NGS: Next generation sequencing
Non-SCC: Non-squamous cell carcinoma
NSCLC: Non-small-cell lung carcinoma
SCC: Squamous cell carcinoma
SPC: Simple processing control
TK: Tyrosine kinase
TKIs: Tyrosine kinase inhibitors
