# Supplementary material for: Clinical performance evaluation of the Idylla™ EGFR Mutation Test on formalin-fixed paraffin-embedded tissue of non-small cell lung cancer
Source: BMC Cancer. 2020 Apr 3;20:275. doi: 10.1186/s12885-020-6697-7 (PMC7126408; doi:10.1186/s12885-020-6697-7)
Supplement: Supplementary file 1 — Additional file 1 Supplementary Table 1. post-CPE study cohort. Supplementary Table 2. age of prepared FFPE blocks. Supplementary Table 3. assessment of the tissue area. Supplementary Table 4. evaluation of necrotic tissue and tar. Supplementary Table 5: post-CPE results. Supplementary Table 6. agreement table at the dichotomous level for valid, non-missing results. Supplementary Table 7. post-CPE measures of agreement. [file 12885_2020_6697_MOESM1_ESM.docx]

SUPPLEMENTARY MATERIAL

**Supplementary table 1:** post**-**CPE study cohort.

| **Post CPE study** | **Positive *EGFR*** | **Negative *EGFR*** | **Total** |
| --- | --- | --- | --- |
|  |  |  |  |
| Sevilla | 0 | 24 | 24 |
|  |  |  |  |
| Bellvitge | 15 | 1 | 16 |
|  |  |  |  |
| Terrasa | 7 | 1 | 8 |
|  |  |  |  |
| Coimbra | 5 | 0 | 5 |
|  |  |  |  |
| **Total** | **27** | **26** | **53** |

**Supplementary table 2:** age of prepared FFPE blocks.

| **Age (years)** | **Valids** | **Invalids** |
| --- | --- | --- |
| <1 | 46 | 4 |
| 1 – 2 | 39 | 3 |
| 2 – 3 | 32 | 5 |
| 3 – 4 | 10 | 1 |
| 4 – 5 | 16 | 0 |
| >5 | 9 | 0 |
| Unknown | 14 | 0 |
| **Total** | **166** | **13** |

No age-related trend regarding invalid was identified. Calculations based on 179 samples tested on Idylla™ initially.

Supplementary table 3: assessment of the tissue area.

Percentage valid results are based on 179 samples tested on Idylla™ initially. (*) Percentage concordant test results are calculated based on the valid test results only. Only true discordant results are taken into account. Discordant results by design are ignored for the calculation of the concordant results.

Supplementary table 4: evaluation of necrotic tissue and tar.

| **Percentage Necrotic tissue in FFPE tissue area** | **Valid (%)** | **Concordant (%)*** |
| --- | --- | --- |
| <10 | 97/104 (93.3) | 66/70 (94.3) |
| 10 – 25 | 19/22 (86.4) | 12/12 (100) |
| 25 -50 | 9/10 (90) | 8/8 (100) |
| >50 | 3/3 (100) | 1/1 (100) |
| Unknown | 38/40 (95) | 29/30 (96.7) |
| **TAR presence in tissue area** |  |  |
| Absent | 65/72 (90.3) | 45/49 (91.8) |
| Present | 63/67 (94) | 43/43 (100) |
| Unknown | 38/40 (95) | 28/29 (96.55) |

Percentage valid results are based on 179 samples tested on Idylla™ initially. (*) Percentage concordant test results are calculated based on the valid test results only. Only true discordant results are taken into account. Discordant results by design are ignored for the calculation of the concordant results.

Supplementary table 5: post-CPE results.

| ^Therascreen®^  Idylla™ | DelEx19 | DelEx19,T790M | L858R | L858R, S768I | G719X,L861Q | L858R,T790M | InsEx20 | WT | **Totals** |
| --- | --- | --- | --- | --- | --- | --- | --- | --- | --- |
| DelEx19 | 10 |  |  |  |  |  |  |  | **10** |
| DelEx19,T790M |  | 2 |  |  |  |  |  |  | **2** |
| L858R |  |  | 9 |  |  | 1 |  |  | **10** |
| L858R, S768I |  |  |  | 1 |  |  |  |  | **1** |
| G719X,L861Q |  |  |  |  | 1 |  |  |  | **1** |
| L858R,T790M |  |  |  |  |  | 1 |  |  | **1** |
| WT | 1 |  | 1 |  |  |  | 1 | 25 | **28** |
| **Totals** | **11** | **2** | **10** | **1** | **1** | **2** | **1** | **25** | **53** |

Supplementary table 6: agreement table at the dichotomous level for valid, non-missing results.

|  | **Therascreen^®^** | | |
| --- | --- | --- | --- |
| **Idylla™** | Mutant | WT | Totals |
| Mutant | 25 | 0 | 25 |
| WT | 3 | 25 | 28 |
| Totals | 28 | 25 | **53** |

Supplementary table 7: post-CPE measures of agreement.

| Measure | Rate | Point estimate | 95% lower limit (1-sided) | 95% upper limit (1-sided) |
| --- | --- | --- | --- | --- |
| Overall Diagnostic Agreement | 50/53 | 94.34 | 84.63 | 98.06 |
| Positive Diagnostic Agreement | 25/28 | 89.26 | 72.80 | 96.29 |
| Negative Diagnostic Agreement | 25/25 | 100 | 86.68 | 100 |
